# FSHD: A Repeat Contraction Disease Finally Ready to Expand (Our Understanding of Its Pathogenesis)

**DOI:** 10.1371/journal.pgen.1001180

**Published:** 2010-10-28

**Authors:** Christopher E. Pearson

**Affiliations:** 1 Program of Genetics and Genome Biology, The Hospital for Sick Children, Toronto, Ontario, Canada; 2 Department of Molecular Genetics, University of Toronto, Toronto, Ontario, Canada; The University of North Carolina at Chapel Hill, United States of America

Facioscapulohumeral muscular dystrophy (FSHD), was one of the first diseases shown to be caused by an unstable repeat in the early 1990s along with spinal and bulbar muscular atrophy (SBMA), myotonic dystrophy (DM1), and fragile X mental retardation (FRAXA), where the latter three are caused by genetically expanding trinucleotide repeats [1]. However, FSHD differs considerably from the trinuclotide repeat diseases, as it is caused by a contraction of a macrosatellite (D4Z4 repeat, 3.3 kb/unit). Moreover, far less is understood about the pathogenic mechanism for FSHD, relative to SBMA, DM1, and FRAXA. This is not due to a shortage of experimental efforts, plausible hypotheses, or collaborative efforts towards understanding FSHD [2], [3]. The elucidation of FSHD is hampered by the size of the unstable repeat, its sequence complexity, the number of repeat units, and the presence of the repeat on Chromosomes 4 and 10, making analysis technically difficult. The difficulty is compounded further by the absence of an obvious gene, transcript, or protein in the unstable or proximal region; in fact, the D4Z4 repeats have been referred to as “junk” DNA or are thought to be a pseudogene, at best. As a result, FSHD has proved to be one of the most complex and challenging genetic diseases to even a glimpse an underlying pathogenic cause for FSHD. Several recent papers, including one in this issue of *PLoS Genetics* [4], have made significant advances that now permit us to expand our understanding of FSHD pathogenesis, a repeat contraction disease.

FSHD presents with weakness of facial muscles, stabilizers of the scapula, or dorsiflexors of the foot. The weakness is progressive with age. Disease severity is highly variable and shows some signs of anticipation, common to other repeat-associated diseases. FSHD is autosomal dominant, characterized by a deletion of D4Z4 repeat units, located in the subtelomere of chromosome 4q35 (Figure 1). Non-affected D4Z4 alleles are polymorphic having 11–100 repeat units; individuals affected with FSHD have 10 or fewer units, but must have at least one unit to show disease, which is now known to be the most telomeric unit. D4Z4 contractions can be inherited or occur as de novo mutations. The contracted D4Z4 repeat arrays show loss of DNA methylation and reduced histone 3 lysine 9 trimethylation, consistent with a more open chromatin structure [5]. The role of the altered chromatin in FSHD pathogenesis is controversial and has been suggested to enhance expression of adjacent genes like FRG1 or ANT1 [6]. More recently, FSHD2-affected individuals that display the altered chromatin but have non-contracted D4Z4 repeats have implicated the derepression of a DUX4 transcript encoded on the D4Z4 repeat units [7], [8]. However, the mechanism through which the altered chromatin at D4Z4 repeats contributes to FSHD remains unclear.

**Figure 1:**
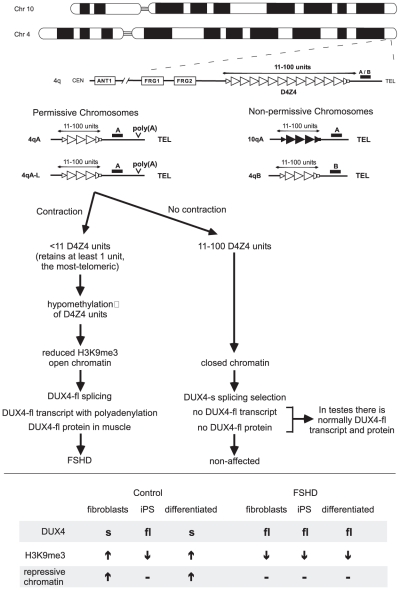
Simplified schematic outlining the genetic requirements for FSHD and the current model for pathogenesis. The Chromosome 4 D4Z4 repeats (open triangles) and its homolog on Chromosome 10 (closed triangles), indicating the 4qA/4qB polymorphisms that define the genetic background of the repeat. Individuals with FSHD have a D4Z4 repeat tract of <11 repeats, at least 1 unit on 4qA but not on 4qB or 10q chromosomes. All permissive chromosomes and FSHD individuals have a distal canonical highly efficient poly(A) motif ATTAAA. Non-permissive chromosomes have inefficient degenerate motifs. Both have alternative poly(A) motifs further downstream. Current model involving contraction, DUX4 transcription, polyadenylation, altered chromatin, regulated DUX4 splicing, tissue- and development-specific DUX-fl protein expression. See text for details. *Lower*, de-differentiation and differentiation affect DUX4-fl expression in control but not FSHD cells. See text for details.

## *DUX4* Transcripts from the D4Z4 Repeats

The distal (most telomeric) unit of the D4Z4 repeat was recently shown to have a transcriptional profile that differs from internal units, and the transcript extended into telomeric regions [9], [10], [11]. This finding suggests that this very last, distal D4Z4 unit may be the key unit that must be retained after D4Z4 contractions to lead to disease (Figure 1). The *DUX4* transcript from the distal D4Z4 unit is suggested to encode a double homeobox gene of unknown function, related to *DUXC* and *Duxbl*, which in mice is expressed in germ-line cells and in early phases of skeletal muscle development. Previously, Tapscott's group showed full-length RNA transcripts from the D4Z4 repeat spanning the *DUX4* open reading frame, DUX4-fl, as well as a shorter transcript, DUX4-s, that utilized a cryptic splice donor, which retains the double-homeobox domains but loses the carboxyterminal end of DUX4 [11]. When expressed in cultured cells, the full length DUX4 caused reduced proliferation, induced morphological changes, increased sensitivity to oxidative damage, MyoD-repression, impaired myogenesis, and, at higher levels, led to cell death—all features compatible with observations in FSHD1 patient cells [11], [12]. This distal DUX4 transcript can be observed in FSHD1 patient myotubes but not in control myotubes [13]. Exactly what mediates the expression in FSHD has proved elusive.

## Genetics Reveals a Role of a Polyadenylation Variant Downstream of the Most-Telomeric Chromomosome 4 D4Z4 Unit

Recent genetic advances made by a group of international labs, headed by Silvere van der Maarel, revealed the most-telomeric D4Z4 unit and its adjacent polyadenylation sequence to be crucial to FSHD pathogenesis [13] (Figure 1). Some Chromosome 4 backgrounds are categorized as permissive for FSHD disease or non-permissive to FSHD when D4Z4 contracts. Chromosome 10 repeats are typically non-permissive. Essentially, D4Z4 contractions to 1–10 units on permissive chromosomes are pathogenic, while contractions on non-permissive chromosomes are non-pathogenic. Sequence comparison of the permissive chromosomes with the common, non-permissive chromosomes failed to reveal a motif unique to the proximal D4Z4 units of the repeat array that might explain the permissiveness of the 4A161 chromosome [13]. Curiously, immediately distal to the most-telomeric D4Z4 unit, there was a polymorphism unique to the permissive 4A161 chromosome. This sequence polymorphism was subsequently shown to act as polyadenylation signal (ATTAAA) of the distal (most telomeric) DUX4 transcript. Notably, all permissive, but not non-permissive, chromosomes harbored the efficient poly(A) signal [9], [13]. This association should be investigated further.

Several rare, but highly informative FSHD individuals were identified that harbored unusual contracted hybrid D4Z4 repeats composed of D4Z4 units from Chromosome 4 and Chromosome 10—some hybrids resided on Chromosome 4, others on Chromosome 10 [13]. Two important conclusions can be made from these individuals. First, the cause of FSHD disease linked to Chromosome 10 excludes a previously suggested role for enhanced expression of the adjacent genes on 4q (FRG1, FRG2, ANT1, etc….) in the pathogenesis of FSHD [6], as these were not present on Chromosome 10. Secondly, in all affected individuals with the unusual hybrid repeats, the last D4Z4 unit of the contracted array originated from a permissive background and had the adjacent highly efficient polyadenylation motif, thereby strengthening the crucial role of this motif to FSHD pathogenesis.

In transfection experiments *DUX4* transcripts derived from the permissive chromosome were stable and efficiently polyadenylated, whereas transcripts derived from non-permissive chromosomes were undetectable and polyadenylation inefficient [13]. Thus, a bona fide poly(A) signal unique to the permissive chromosomes produced stable transcripts with greater polyadenylation efficiency than non-permissive chromosomes, thereby providing strong evidence suggesting that increased polyadenylation, and hence stability, of the distal (most telomeric) *DUX4* transcript may be causally implicated in FSHD pathogenesis (Figure 1). Despite lacking the polyadenylation motif that is present only on the FSHD permissive chromosome, testis (but not muscle cells) are able to express and stably retain the DUX4 transcript. Snider et al. [4] revealed that alternative polyadenylation arises at motifs more telomeric on the non-permissive and permissive chromosomes in testis, but not somatic cells. They suggest that this DUX4 alternative polyadenylation may be regulated in a tissue- and development-specific manner. This important advance revealed that DUX4 transcript and protein was naturally expressed and provided insight into how this expression was regulated, as well as avenues to how it could be misregulated in disease states. Analysis of a broader range of tissues and developmental stages will reveal insight into the function of DUX4.

## Epigenetics May Regulate *DUX4* Splicing

The full-length RNA transcripts from the D4Z4 repeat spanning the *DUX4* open reading frame, DUX4-fl, and the shorter transcript, DUX4-s that utilized a cryptic splice donor produce full-length and truncated DUX4 proteins, respectively [11]. The presence of the full-length DUX4-fl mRNA in control human testes, but not in control muscle, which express the shorter DUX4-s mRNA, was shown by Snider et al. to be mediated by DUX4 splice site usage in this issue of *PLoS Genetics* [4]. Some FSHD samples also expressed DUX4-s transcript. Thus, both control and FSHD myoblasts and muscles transcribe *DUX4*, but the full-length DUXF-fl transcript is expressed only in FSHD cells and muscles. Expression of DUX4-fl, but not DUX4-s, can lead to both nuclear foci and increased apoptosis [4]. However, overexpression of either DUX4-fl or DUX4-s will suppress myogenesis [11], [12]. Understanding the natural function of the DUX4-fl protein and the DUX4-s protein, presuming its careful regulation supports a function, is now a pressing issue in FSHD research. Curiously, the relatively high levels of *DUX4* expression in FSHD cells appears to be due to large numbers of transcripts produced in a small subset of cells, rather than a small number of transcripts produced in most cells. Understanding what regulates non-expression or expression in cells may provide insight into disease pathogenesis and possibly avenues for therapeutically down-regulating *DUX4* expression in FSHD individuals. Similar insights may arise from understanding what regulates the splicing of the DUX4 transcript.

Snider et al. [4] suggest that DUX4 splice site usage may be regulated by epigenetic modifications of the D4Z4 region (Figure 1). Control fibroblasts expressed DUX4-s, but not DUX4-fl; however, when these cells were made into induced pluripotent stem cells (iPS) they switched to expression of DUX4-fl (Figure 1, lower portion). Upon differentiation of these control iPS cells, expression switched back from DUX4-fl to DUX4-s. This switch correlated with increased levels of repressive chromatin modification (H3K9me3) at the D4Z4 repeats in the differentiated cells. In contrast, in FSHD fibroblasts, their iPS derived cells, and their differentiated stages, DUX4-fl was consistently expressed. Thus, it appears that higher levels of repressive chromatin at the D4Z4 region in control cells may mediate the splicing that produces DUX4-s. Similarly, the reduced levels of repressive chromatin present in FSHD cells [5] may suppress the production of DUX4-s and allow expression of DUX4-fl.

In conclusion, D4Z4 repeats are not junk DNA: *DUX4* encoded in the repeats is in fact a retrogene, most likely retrotransposed to a DNA gene from the DUXC mRNA [10]. Tapscott and colleagues have shown *DUX4* is normally expressed in the male germline, and for the FSHD-permissive variant, the DUX4 transcript is efficiently polyadenylated in muscle, thus suggesting that *DUX4* is not a “defective” pseudogene. Importantly, the aberrant suppression of DUX4-fl protein expression in muscle, both by transcript stabilization via disease-permissive polyadenylation and suppressed splicing to the shortened DUX4-s variant, seems to be important contributors to FSHD pathogenesis. The mechanisms through which the DUX4 transcripts and proteins lead to normal development or FSHD are future goals waiting to be expanded upon in this macrosatellite contracting disease.
